# Distinguishing Ewing sarcoma and osteomyelitis using FTIR spectroscopy

**DOI:** 10.1038/s41598-018-33470-3

**Published:** 2018-10-10

**Authors:** Radosław Chaber, Christopher J. Arthur, Joanna Depciuch, Kornelia Łach, Anna Raciborska, Elżbieta Michalak, Józef Cebulski

**Affiliations:** 1 Clinic of Paediatric Oncology and Haematology, Faculty of Medicine, University of Rzeszow, Rzeszow, Poland; 2 School of Chemistry, University of Bristol, Bristol, United Kingdom; 3 Institute of Nuclear Physics Polish Academy of Sciences, Krakow, Poland; 4 Department of Oncology and Surgical Oncology for Children and Youth, Institute of Mother and Child, Warsaw, Poland; 5 Department of Pathology, Institute of Mother and Child, Warsaw, Poland; 6 Centre for Innovation and Transfer of Natural Sciences and Engineering Knowledge, University of Rzeszow, Rzeszow, Poland

## Abstract

The differential diagnosis of Ewing sarcoma and osteomyelitis can be challenging and can lead to delays in treatment with possibly devastating results. In this retrospective, small-cohort study we demonstrate, that the Fourier Transformed Infrared (FTIR) spectra of osteomyelitis bone tissue can be differentiated from Ewing sarcoma and normal bone tissue sampled outside tumour area. Significant differences in osteomyelitis samples can be seen in lipid and protein composition. Supervised learning using a quadratic discriminant analysis classifier was able to differentiate the osteomyelitis samples with high accuracy. FTIR spectroscopy, alongside routine radiological and histopathological methods, may offer an additional tool for the differential diagnosis of osteomyelitis and ES.

## Introduction

Ewing sarcoma (ES) is a poorly differentiated tumour of bones or soft tissues derived from primitive mesenchymal stem cells. This tumour is the second most common bone malignancy with 2.9 cases per million population (below the age of 20). With advances in multimodal therapy, survival rates for patients with localized disease approach 70%. Patients with metastatic, refractory, or relapsed disease, however, have a poor outcome (5-yrs. overall survival about 42%)^1^. Unfortunately, misdiagnosis of Ewing Sarcoma and the bone disease osteomyelitis is well-reported due to the similarities in their presentation. In both delayed or misdiagnosis can lead to adverse clinical outcomes.

Osteomyelitis arises because of infection of the bone with bacteria being the usual etiologic agents (most commonly *Staphylococcus aureus*). In children, osteomyelitis is primarily of haematogenous origin, occurring less commonly as a result of trauma, surgery, or infected contiguous soft tissue^2^. Acute osteomyelitis affects about 8 children per 100,000 children/year^3^. Children under 5 years of age are affected in about 50% of the cases, with a male/female ratio of 2:1. Acute osteomyelitis is approximately twice as common as septic arthritis, and its incidence is steadily increasing (due in part to the growing frequency of antibiotic resistance amongst *Staphylococci*)^3^.

The similarity of the clinical course and the radiological and histopathological picture of ES and osteomyelitis makes it difficult to distinguish these two diseases objectively. Although open biopsy may provide a better diagnostic yield, it has limitations, particularly in patients with ES. McCarville *et al*. suggest that when an open biopsy is not diagnostic, such patients are more likely to harbour a malignancy than an infection and it is thus recommended to perform a repeat biopsy to initiate appropriate therapy^4^. Given the challenge of differentiating these two conditions, it is essential that new diagnostic methods can be discovered that can aid diagnosis in cases where the diagnosis is in doubt.

Fourier Transform Infrared (FTIR) spectroscopy has emerged in recent years as a promising tool for the study and diagnosis of disease. FTIR measures the absorption of infrared radiation by the chemical bonds in constituent molecules of a sample. It therefore provides bulk information about the biochemical composition of a sample as opposed to immunohistochemistry or other molecular techniques that selectively target specific macromolecule types^5^. Despite this lack of selectivity, FTIR is advantageous due to its small sample size (a few micrograms) and the limited requirements on the sample (tissue) pre-processing (e.g., demineralization, staining, deparrafinization)^6^. As such, FTIR spectroscopy can be applied to early stages of a disease, before tissue changes are detectable by light microscopy. FTIR has also been used to monitor disease course and therapeutic outcome^7^. In addition to this clinical utility, FTIR spectroscopy is rapid, inexpensive and straightforward to perform. The FTIR features of Ewing sarcoma infected bone tissue have been previously reported^8^, however to-date no study has reported the FTIR spectroscopic features of osteomyelitis nor compared those with Ewing Sarcoma to determine whether they differ.

In view of the importance of misdiagnosis of osteomyelitis and ES on patient outcomes we sought to investigate the diagnostic potential of FTIR spectroscopy in differentiating these two clinically important diseases. To that end we report the analysis of bone tissue from patients with ES or osteomyelitis using ATR-FTIR and statistical methods.

## Materials and Methods

### Patients

Samples of bone tissue were obtained from 27 patients with ES (aged 5–20 years old with a median age of 14, male/female ratio of 12/15) and 10 patients with osteomyelitis (age 2–17 years old with a median age of 11 years and a male/female ratio of 6/4). Samples were allocated to three study groups: group I – normal bone tissue sampled outside the area of ES infiltration after neoadjuvant chemotherapy completion (20 samples), group II – osteomyelitis bone tissues (10 samples) and 27 ES bone tissues collected during a diagnostic biopsy (group III). The samples in the first group were collected from ES patients. Surgery was performed at the Department of Surgical Oncology, Institute of Mother and Child in Warsaw, Poland. Each histopathological sample was verified at the same institution by pathologists experienced in bone disorders.

The study was conducted under Institutional Review Board (Protocol No. KBET/6/06/2014) from June 2014 at University of Rzeszow. The experimental protocols used in this study were approved by the institutional ethics committees (IECs) of the University of Rzeszow and were carried out in accordance with the approved guidelines. Informed consent was obtained from all patients or their guardians before treatment.

### Samples preparation for FTIR measurements

Samples were prepared for FTIR measurement according to the procedure of Depciuch *et al*.^9^. The bone tissue specimens were placed for about 12 h in liquid fixative. Afterwards, the ethanol content within the tissue was gradually replaced with xylene, starting from 50%, then 70%, 80%, 90% and 96%, up to anhydrous (absolute 99.8% of xylene). Then the samples were rinsed with distilled water and dried. Each incubation step was performed for 5 min. Such Formalin-fixed paraffin-embedded (FFPE) bone tissue specimens were then cut into 10 μm thick sections using a rotary microtome. FFPE tissue sections were then applied to calcium fluoride (CaF_2_) slides.

### FTIR Spectroscopy

FTIR spectra were recorded using a Vertex 70v Fourier transform infrared spectrometer (Bruker). Tissue specimens were directly applied to the attenuated total reflection (ATR) diamond crystal and spectra in the mid-infrared range were recorded (32 scans with 2 cm^−1^ of spectral resolution). As the samples were dewaxed, the air was measured as the background. All measurements were recorded in triplicate. Data analysis was performed using the program OPUS 7.0 from Bruker Optik GmbH 2011. Spectra were mean centred, scaled to unit variance, smoothed using a Savitzky–Golay filter and a linear detrend applied.

### Deconvolution of amide I region (1600–1700 cm^−1^)

Protein secondary structure was analysed by means of curve fitting using MagicPlot 2.7.2. First, the second derivative spectra were determined based on the ATR-FTIR spectra to determine the initial peak positions for curve fitting, and the peaks were fitted using a Gaussian function. The sum of value of all maxima absorbance corresponding to α-helix and β-sheet was considered 100% and each component (separately for α-helix and β-sheet) was expressed as its percentage after fitting.

### Statistics

The data were analysed using the one-way ANOVA followed by the Tukey’s test. A p-value < 0.05 was considered statistically significant with the 95% confidence interval (MatLab).

### Data analysis

For all obtained spectra, vector normalization and baseline correction were applied. These operations were performed using OPUS 7.0 software. Moreover, in each FTIR spectrum, a vibrations corresponding to nucleic acid, phospholipids, proteins, lipids, were analysed. The number of obtained data from FTIR therefore, to determine a similarity between analysed three groups, a PCA analysis, was done. PCA reduces the dimensionality, the number of variables of the data, by maintaining as much variance as possible. This analysis was done using Past software. Moreover, to determine the similarity between samples within the groups, as well as between I, II and III groups, a hierarchical cluster analysis (HCA) using Past software, were done. Further data analysis including clustering and dimensionality reduction was performed using Python 3.6 and Scikit Learn 0.19.1.

### Compliance with ethical standards

All procedures performed in studies involving human participants were in accordance with the ethical standards of the institutional and/or national research committee and with the 1964 Helsinki declaration and its later amendments or comparable ethical standards. The study was approved by the Committee for Research Ethics of University of Rzeszow (decision 6/06/2014).

### Approval

All experimental protocols were approved by a named institutional.

### Accordance

The methods were carried out in accordance with the relevant guidelines and regulations. Informed consent was obtained from all individual participants included in the study. All data is availability.

## Results

**Figure 1:**
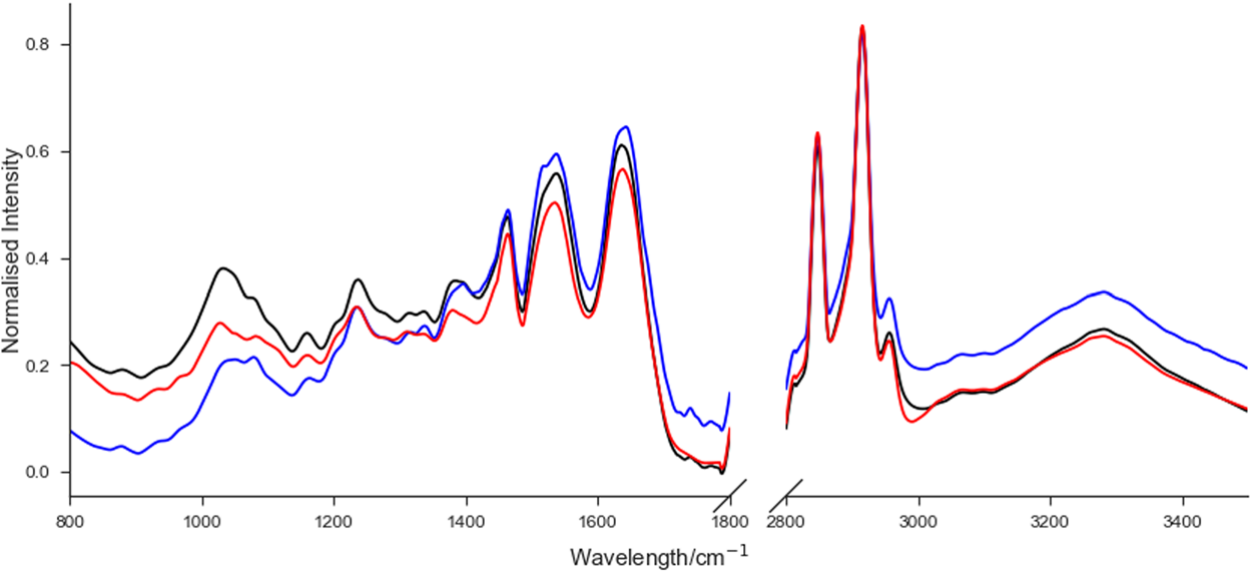
Normalised average FTIR spectra of normal bone tissue sampled outside the area of ES infiltration (black), osteomyelitis bone tissue (blue) and ES bone tissue (red).

As expected, peaks corresponding to functional groups within nucleic acids, phospholipids, polysaccharides, proteins and lipids are observable in the FTIR spectra of bone tissues (Fig. 1). The peak positions along with their maximum absorbance for the average FTIR spectra with corresponding vibrations are described in Table 1.

**Table 1:** FTIR peaks position with corresponding vibrations in analysed three groups^23–29^.

FTIR spectroscopy peaks (cm^−1^)						
normal bone tissue sampled outside the area of ES infiltration		Osteomyelitis bone tissue		ES bone tissue		Assignment
Peaks	Max. (a.u.)	Peaks	Max. (a.u.)	Peaks	Max. (a.u.)	
1029	0.421	1041	0.992	1059	0.041	PO_3_^−2^ group from DNA, RNA and phospholipids^23^
1162	0.074	1170	0.011	1169	0.012	C-O group from groups of serine, threonine, and tyrosine of protein^24^
1234	0.312	1250	0.031	1238	0.312	Amide III^25^
1337	0.008	1320	0.008	1342	0.011	CH_2_ group from protein^25^
1396	0.010	1380	0.010	1382	0.012	Possible carbonate band ʋ_2_ (CO_3_^2−^)^23^
1462	1.412	1465	1.413	1467	1.412	CH_2_ group from cholesterol^23^
1540	1.761	1542	1.932	1551	1.763	Amide II^26^
1635	2.000	1649	2.102	1657	2.000	Amide I^26^
1740	−1.251	1740	−0.091	1740	−1.251	C=O stretching vibrations from aldehyde^27^
2848	2.402	2858	2.102	2848	2.913	symmetric stretching vibrations of CH_2_^27^
2916	4.098	2910	3.213	2906	4.201	asymmetric stretching vibrations of CH_2_^27^
2956	0.123	2958	0.123	2958	0.123	asymmetric stretching vibrations of CH3^27^
3283	0.013	3295	0.003	3289	0.012	ʋ-NH stretching of the peptide bond (-NHCO) of proteins^28^ and ʋ-OH stretching of functional groups of water^29^

Visible shifts of the peaks between osteomyelitis and ES bone tissue spectra compared to normal bone tissue sampled outside the area of ES infiltration could be observed. Moreover, shifts of the peaks between osteomyelitis and ES bone tissue could also be seen. Moreover, the changes in the FTIR range corresponding to amide II vibrations, were significantly different between osteomyelitis and ES bone tissue as well as normal bone tissue sampled outside the area of ES infiltration.

The maximal absorbance values of individuals are similar in each group, whilst between groups differences can be discerned. The most visible differences in the values of absorbance in wavenumbers corresponding to DNA, phospholipids, amide II and lipids, were observed. The highest value of absorbance of peak at 1029 cm^−1^ in the FTIR spectrum of normal bone tissue sampled outside the area of ES infiltration, were observed, while the lowest value of absorbance of this peak in the FTIR spectrum of osteomyelitis bone tissue, were visible. In the in the FTIR spectrum of osteomyelitis tissue, however, the absorbance at 1540 cm^−1^, was greatest, whilst in the FTIR spectra of the ES bone tissue, the highest value of peaks at 2848 cm^−1^, 2916 cm^−1^, 2956 cm^−1^, were observed.

Deconvolution of the amide I region allows information about the type of secondary structure to be obtained, as well as the changes in the values of α-helix and β-sheet in proteins to be monitored (Fig. 3). The peaks between 1600–1640 cm^−1^ and between 1670–1700 cm^−1^ corresponding to the secondary structure of proteins vibrations of β-sheet and β-turn, respectively. Furthermore, the IR range around 1650 cm^−1^ originates from α-helix^10^.

**Figure 2:**
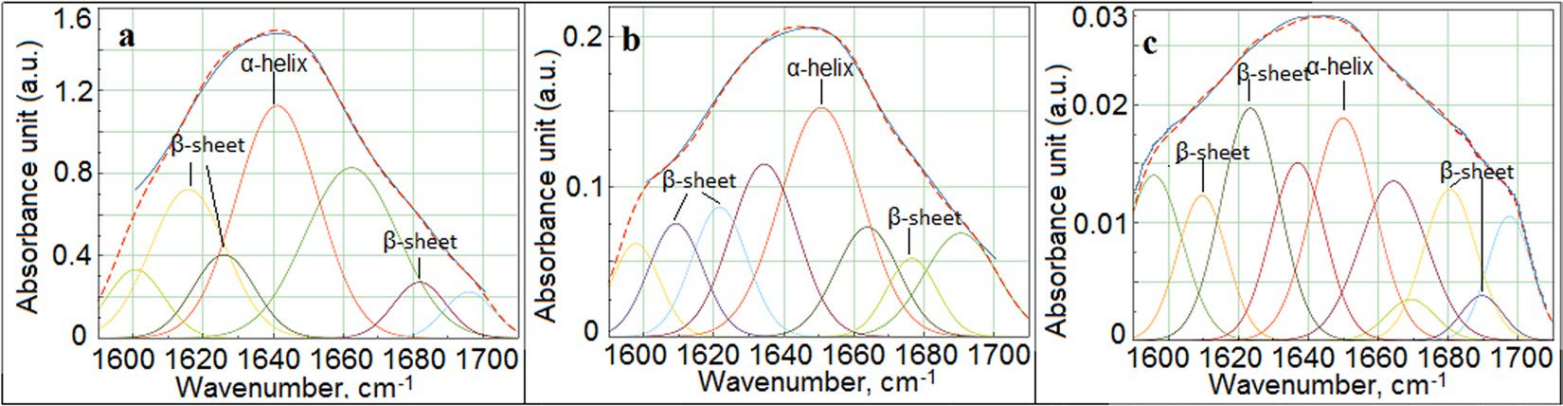
Deconvolution of amide I FTIR region (1700–1600 cm^−1^) obtained for three analysed groups: normal bone tissue sampled outside the area of ES infiltration (**a**) ES bone tissue (**b**) osteomyelitis bone tissue (**c**). The peaks for calculated α-helix - β-sheet ratio are indicated.

A different number of band components are observable in the three analysed groups (Fig. 2): with seven band components in the normal bone tissue, eight in the ES bone tissue and ten components in the osteomyelitis bone tissues. In the next step, the α-helix - β-sheet ratio was calculated from the deconvolution of amide I FTIR range (Table 2). The ratio of secondary protein structure is significantly lower in ES and osteomyelitis bone tissues compared to normal bone tissue. Moreover, the calculated ratio for ES bone tissue and osteomyelitis bone tissues, also differed but without statistical significance.

**Table 2:** Maximum absorbance values for α-helix and β-sheet, percentage and ratio of protein α and β secondary structures.

Sample	Value of maximum absorbance		Percentage [%]		α-helix - β-sheet ratio
	α-helix	β-sheet	α-helix	β-sheet	
normal bone tissue sampled outside the area of ES infiltration	1.11 ± 0.52	1.39 ± 0.43	44.4 ± 47.31	55.6 ± 30.94	0.80 ± 0.02
ES bone tissue	0.15 ± 0.03*	0.21 ± 0.07*	41.66 ± 20.00	58.44 ± 33.33	0.71 ± 0.06*
Osteomyelitis bone tissue	0.02 ± 0.00*	0.05 ± 0.00*	28.57 ± 0.00*	71.43 ± 0.00*	0.78 ± 0.00*

Value of peaks area, percentage and ratio of protein α and β secondary structures.

*p < 0.05, vs. normal bone tissue sampled outside the area of ES infiltration.

**Figure 3:**
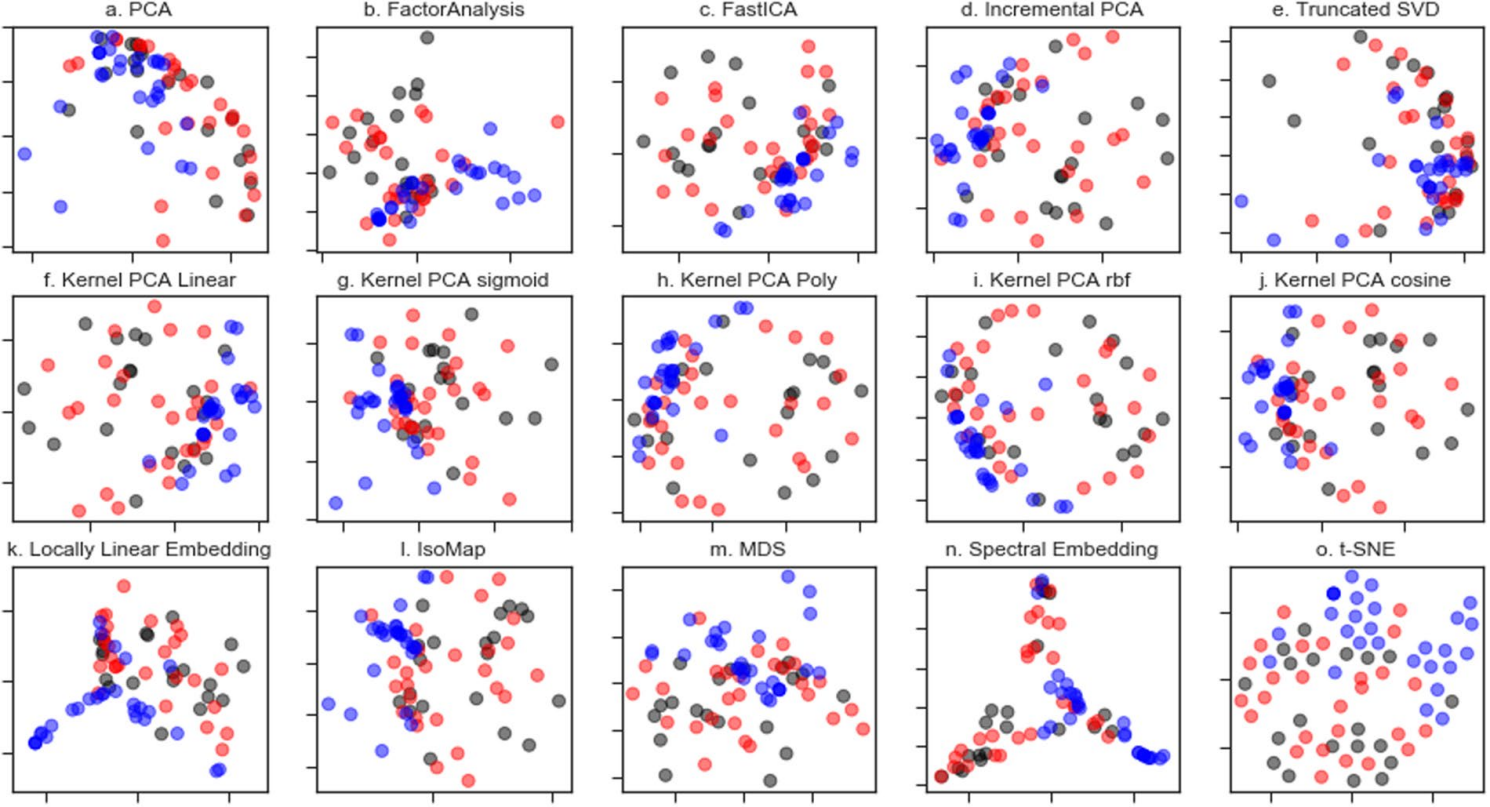
Dimensionality reduction methods applied to the FTIR dataset. Matrix decomposition methods (**a**). PCA (**b**). Factor analysis (**c**). Fast Independent Components Analysis (FastICA) (**d**). Incremental PCA (**e**). Truncated singular value decomposition (SVD) (**f**). Kernel PCA using a linear kernel. (**g**) Kernel PCA using a sigmoid kernel. (**h**). Kernel PCA using a polynomial kernel. (**i**). Kernel PCA using a radial basis function kernel. (**j**). Kernel PCA using a cosine kernel. Manifold Learning methods (**k**). Locally linear embedding. (**l**) Isomap (**m**). Multidimensional scaling (MDS) (**n**). Spectral embedding and (**o**). t-distributed stochastic neighbour embedding (t-SNE).

We then turned our attention to whether it is possible to differentiate the ES samples from the osteomyelitis samples and turned in the first instance to unsupervised dimensionality reduction *via* principal components analysis (PCA). Dimensionality reduction is broadly based on the selection of the informative features or on the synthesis of variables that retain the information present in the original dataset. In principal component analysis this dimensionality reduction is achieved by finding the linear combination of a set of variables that has maximum variance. When the spectral dataset was analysed by PCA (Fig. 3a), although the osteomyelitis samples (blue) clustered together to some extent there was considerable overlap between all three tissue classes.

More recent methods for dimensionality reduction, particularly non-linear (manifold) methods, have emerged which we speculated may allow the tissue classes to be differentiable. A suite of dimensionality reduction methods, including both matrix deconvolution and manifold learning methods as implemented in the python library Scikit Learn, were applied (as shown in Fig. 3). Many of these methods afforded greater visual separation of the classes than PCA. Of note is t-distributed stochastic neighbour embedding (t-SNE, Fig. 3o), which resulted in clear clustering of the osteomyelitis bone spectra. All methods struggled to discriminate between the normal and ES samples although t-SNE shows some clustering of the ES samples into two apparent sub-groups. From Fig. 3 normal bone and ES tumour bone are poorly differentiated by the first two components for each method alone. We interpret this as there being fewer bulk changes to the biochemical composition of the Ewing Sarcoma tumour than that observed in the infected Osteomyletis bone.

Although the unsupervised dimensionality reduction revealed poor differentiation in the first two components of the samples (particularly of the normal and ES tumour), we speculated that higher order components, and combinations thereof, may allow the differentiation of the relative classes in supervised classification. We thus worked towards producing a predictive model capable of distinguishing the tissue classes and thus turned to supervised learning based classification. Supervised learning is the task of inferring a class from a set of labelled training data by fitting to a model that can predict the class of unknown labels of other objects (the test set). Stratified sampling was used to split the dataset into a training (67%) and test set (33%) maintaining the same ratio of class samples. Given the high dimensionality of the IR data set we first generated new features from the dimensionally reduced representations from PCA, Kernel PCA (sigmoid), isomap and factor analysis and stacked these together to generate a new reduced feature set. Training set data were then mapped onto these new dimensionality reduced spaces. Taking the dimensionally reduced versions of the spectra we then trained machine learning models in the hopes of generating a predictive model able to differentiate tissue classes. As the most effective algorithm for a classification task cannot be discerned *a priori* the following classifiers were assessed: nearest neighbours, support vector machine with a linear kernel, support vector machine with a radial basis function kernel, Gaussian Process, decision tree, random forest, neural network, AdaBoost, Naive Bayes, quadratic discriminant analysis, linear discriminant analysis, Nu-Support Vector classification, and Gradient Boosted classifier. Each was used as implemented using Scikit-Learn.

**Figure 4:**
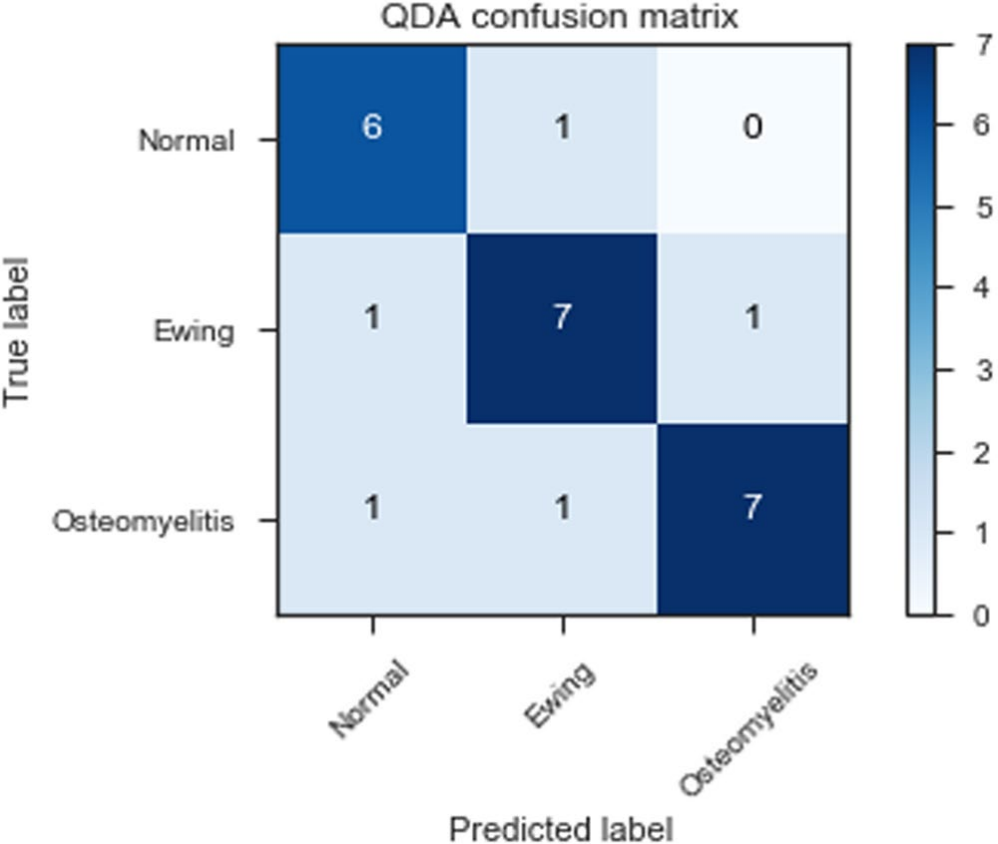
Confusion matrix of a QDA classifier applied to the dimensional reduced spectral data as validated against an external test set.

Through this analysis we identified a quadratic discriminant analysis (QDA) classifier as having the best discriminating power against the external test set (80%) (see Table 3 for precision, recall and f1-scores for this classifier). QDA is a machine learning method which uses quadratic surface to separate classes from one another to produce a prediction model. Significantly this classification model was able to identify osteomyelitis with an 88% precision and 78% recall. The confusion matrix for this classifier is shown in Fig. 4. In comparison, the related random forest method had an lower accuracy compared to the QDA classifier in leave-one-out cross validation (60%) and had a poorer performance against the external test set (68%). In particular the random forest classifier struggled discerning normal and ES tissue from one another. Most classifiers struggled to discern the normal and Ewing sarcoma tissue sections from one another based on the dimensionality-reduced representations alone.

**Table 3:** Precision, recall and f1-score from the QDA classifier used in this work.

	precision	recall	f1-score
Normal	0.78	0.78	0.78
Ewing Sarcoma	0.75	0.86	0.80
Osteomyelitis	0.88	0.78	0.82

## Discussion

**Figure 5:**
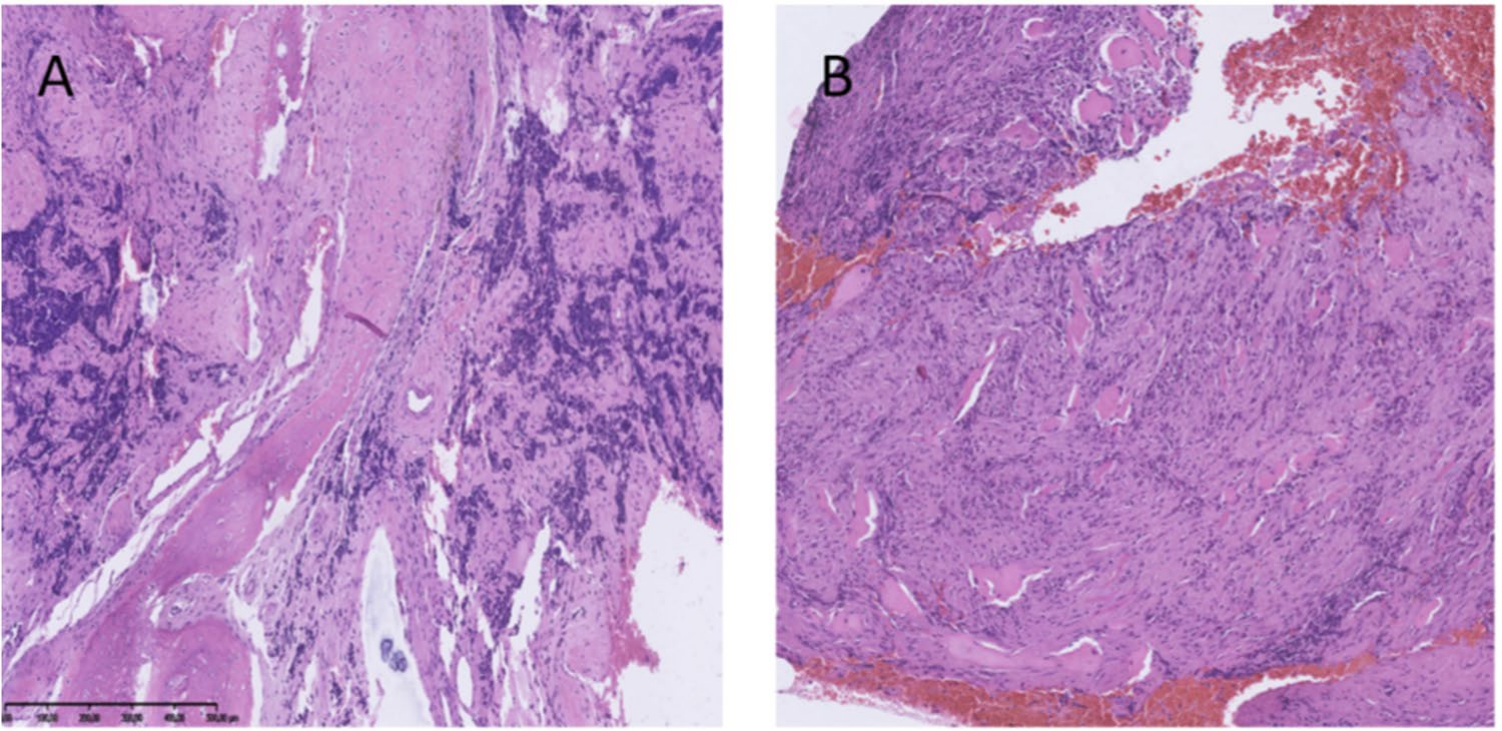
Ewing sarcoma (**A**) and osteomyelitis (**B**) in the light microscopy. Hematoxylin and eosin stained. *E. Michalak*.

Osteomyelitis is a disease caused by the development of inflammatory reaction in bone and bone marrow tissue. The inflammation causes a specific or non-specific disturbance of the physicochemical balance in cells and affected tissues. It remains one of the most difficult to cure infectious diseases^11^. Unfortunately, the clinical, radiological and pathological (Fig. 5) presentation of osteomyelitis can be very similar to malignant ES symptoms^4,12,13^.

Therefore, new methods for distinguishing osteomyelitis bone tissue and ES bone tissues are needed. The emerging diagnostic applications of FTIR spectroscopy are growing as the field matures and it has been applied successfully to the study of cancer^14–18^.

In this study FTIR spectroscopy was used to determine the chemical composition in normal, osteomyelitis and ES bone tissue. We observed the biggest differences in the peaks positions and the values of maximum absorbance in the osteomyelitis samples compared to normal and ES samples (Figs 1 and 2, Table 1). It reflects the meaningful qualitative and quantitative differences in chemical composition between osteomyelitis tissue and other analysed tissues, but some differences were also observed between ES and normal bone tissues. The highest differences in osteomyelitis group were related to the values of maximum absorbance in the FTIR region corresponded to lipids functional groups (Figs 1 and 2, Table 1). The altered lipid content in this group, among other reasons, can be a result of increased concentration of lipid hydroperoxide (LOOH). This enzyme is one of the markers of oxidative stress^19,20^ and is responsible for the oxidative degradation of lipids to reactive aldehydes such as malondialdehyde and 4-hydroxynonenal^21^. The values of absorbance for aldehyde group at wavenumbers 1740 cm^−1^ in FTIR spectra of osteomyelitis bone tissues are higher than the values of these peaks in other two groups. This is consistent with the presence of an active lipid peroxidation related to the inflammatory process in osteomyelitis affected bone. Moreover, changes in the chemical local microenvironment are dependent upon cell proliferative activity and cell cycle phase^22^, which may also explain some of the observed differences in lipid and protein contents seen in the analysed sample groups.

The different absorbance values for the FTIR region corresponding to amide II and amide I groups in the normal bone samples compared to ES and osteomyelitis bone tissues samples reflect the qualitative and quantitative dissimilarity in protein content between them. The deconvolution of the amide I FTIR region (Fig. 2) revealed the different number of bands compounds in the each of analysed bone samples. Furthermore, the α-helix/β-sheet ratio (Table 2) shows, that the secondary structure of proteins in normal bone tissue differs from that in the ES and osteomyelitis bone tissue. The highest value of α-helix - β-sheet ratio in normal bone tissue is significantly different than corresponding ratio value in ES and osteomyelitis bone samples (Table 2). The different protein profile in the analysed three classes of bone tissue is related to the different cellular and tissue composition of them. The common feature in osteomyelitis and Ewing sarcoma is an inflammatory infiltration of immunological cells such as lymphocytes, neutrophils, monocytes, platelets and others. This infiltration determines the different protein content in ES and osteomyelitis samples compare to normal bone tissue.

When a diagnosis error leads to incorrect or delayed treatment, or indeed no treatment at all, a patient’s condition can be worsened. The differential diagnosis of Ewing Sarcoma and osteomyelitis is challenging and misdiagnosis is well-known and can lead to adverse clinical outcomes. Early detection is crucial not only for ES diagnosis, where the delay of treatment may result in tumour dissemination with a significant worsening of prognosis, but also for osteomyelitis, where a delay of only 4 days in the diagnosis is a risk factor for long-term sequelae^3^. The application of FTIR spectroscopy to the study of malignant bone tumours and other bone pathologies remains unexplored. Given the natural, low incidence of both ES and osteomyelitis this work is limited by both its number of subjects including and retrospective character. Our findings, however, suggest that FTIR spectroscopy combined with dimensionality reduction and machine learning methods can distinguish osteomyelitis bone tissue, Ewing Sarcoma and normal bone tissue from one another with a high degree of accuracy.

## Conclusions

Supervised learning using a gradient boosted classifier was able to differentiate the Ewing sarcoma and osteomyelitis samples from one another with high accuracy. We propose, based on this small cohort study, that FTIR spectroscopy, alongside routine radiological and histopathological methods, may offer an additional, powerful and straightforward tool to distinguish ES and osteomyelitis. We are working to confirm the diagnostic in further, prospective studies with more numerous groups of patients.
